# Porcine Small Intestinal Submucosa (SIS) as a Suitable Scaffold for the Creation of a Tissue-Engineered Urinary Conduit: Decellularization, Biomechanical and Biocompatibility Characterization Using New Approaches

**DOI:** 10.3390/ijms23052826

**Published:** 2022-03-04

**Authors:** Martina Casarin, Tiago Moderno Fortunato, Saima Imran, Martina Todesco, Deborah Sandrin, Giulia Borile, Ilaria Toniolo, Massimo Marchesan, Gino Gerosa, Andrea Bagno, Filippo Romanato, Emanuele Luigi Carniel, Alessandro Morlacco, Fabrizio Dal Moro

**Affiliations:** 1 Department of Surgery, Oncology and Gastroenterology, Giustiniani 2, 35128 Padua, Italy; martina.casarin@unipd.it (M.C.); fabrizio.dalmoro@unipd.it (F.D.M.); 2 L.i.f.e.L.a.b. Program, Consorzio per la Ricerca Sanitaria (CORIS), Veneto Region, via N. Giustiniani 2, 35128 Padua, Italy; fortunato.tiago@gmail.com (T.M.F.); saima.imran@unipd.it (S.I.); martina.todesco@unipd.it (M.T.); sandrin.deborah@gmail.com (D.S.); gino.gerosa@unipd.it (G.G.); andrea.bagno@unipd.it (A.B.); filippo.romanato@unipd.it (F.R.); 3 Department of Cardiac, Thoracic Vascular Sciences and Public Health, University of Padua, via Giustiniani 2, 35128 Padua, Italy; 4 Department of Industrial Engineering, University of Padua, via Marzolo 9, 35131 Padua, Italy; ilaria.toniolo.1@phd.unipd.it (I.T.); emanueleluigi.carniel@unipd.it (E.L.C.); 5 Department of Physics and Astronomy ‘G. Galilei’, University of Padova, via Marzolo 8, 35131 Padua, Italy; giulia.borile@unipd.it; 6 Laboratory of Optics and Bioimaging, Institute of Pediatric Research Città della Speranza, 35127 Padua, Italy; 7 Consultant of Animal Welfare and Food Inspection, 35100 Padua, Italy; massimo_marchesan@yahoo.it

**Keywords:** small intestinal submucosa, decellularization, urinary diversions, regenerative medicine, tissue engineering, biomaterial

## Abstract

Bladder cancer (BC) is among the most common malignancies in the world and a relevant cause of cancer mortality. BC is one of the most frequent causes for bladder removal through radical cystectomy, the gold-standard treatment for localized muscle-invasive and some cases of high-risk, non-muscle-invasive bladder cancer. In order to restore urinary functionality, an autologous intestinal segment has to be used to create a urinary diversion. However, several complications are associated with bowel-tract removal, affecting patients’ quality of life. The present study project aims to develop a bio-engineered material to simplify this surgical procedure, avoiding related surgical complications and improving patients’ quality of life. The main novelty of such a therapeutic approach is the decellularization of a porcine small intestinal submucosa (SIS) conduit to replace the autologous intestinal segment currently used as urinary diversion after radical cystectomy, while avoiding an immune rejection. Here, we performed a preliminary evaluation of this acellular product by developing a novel decellularization process based on an environmentally friendly, mild detergent, i.e., Tergitol, to replace the recently declared toxic Triton X-100. Treatment efficacy was evaluated through histology, DNA, hydroxyproline and elastin quantification, mechanical and insufflation tests, two-photon microscopy, FTIR analysis, and cytocompatibility tests. The optimized decellularization protocol is effective in removing cells, including DNA content, from the porcine SIS, while preserving the integrity of the extracellular matrix despite an increase in stiffness. An effective sterilization protocol was found, and cytocompatibility of treated SIS was demonstrated from day 1 to day 7, during which human fibroblasts were able to increase in number and strongly organize along tissue fibres. Taken together, this in vitro study suggests that SIS is a suitable candidate for use in urinary diversions in place of autologous intestinal segments, considering the optimal results of decellularization and cell proliferation. Further efforts should be undertaken in order to improve SIS conduit patency and impermeability to realize a future viable substitute.

## 1. Introduction

Bladder cancer is among the most common malignancies in the world (10th in rank) and at an almost-similar level as a cause of mortality [1]. It is classified as superficial in the case of non-muscle invasive bladder cancer, and as muscle-invasive 1 in the case of advanced stages. Even if 70% of bladder cancers are non-muscle-invasive and can be treated only by transurethral resection of the bladder in the case of low-risk 1, in case of intermediate- and high-risk 1, standards of care recommend the transurethral resection of the bladder, with or without bacillus Calmette–Guerin immunotherapy or chemotherapy [2]. Conversely, in the case of muscle-invasive urothelial bladder cancer (UBC), the standard treatment consists of a radical cystectomy (RC), followed by the creation of a urinary diversion using a segment of the small intestinal tract of the patient. The most commonly observed post-operative complications are associated with the bowel tract removal, including strictures, gastrointestinal (GI) tract bleeding, bowel leakage, fistula formations, and the development of metabolic disorders, depending on the intestinal segment used, the length, and the type of diversion [3,4,5,6,7].

Traditional allotransplantation is limited due to the scarcity of available tissues and organs. In urology, there is an increasing demand for an alternative urinary conduit after radical cystectomy, which can be represented by tissue-engineered urinary conduits instead of autologous intestine. This approach is intended to largely improve the surgical procedure, limiting its duration and reducing possible intra-operatory risks, but it can also decrease patients’ post-operative complications (e.g., stone formation, recurrent urinary tract infections, uretero-enteric strictures, renal function deterioration, and incontinence) [8,9].

Up to now, several types of scaffolds have been investigated [10,11,12,13,14,15,16], obtained either from synthetic or biological materials, in order to find the best replacement device for urinary conduits. Each tested material exhibits its own advantages and limitations: Synthetic materials, such as polyglycolic acid and polylactic-glycolic acid are in use with defined and reliable mechanical features, but they exhibit limited biocompatibility, often eliciting an immune reaction from the host [17,18,19,20,21]. On the other hand, in case of naturally derived polymers (e.g., collagen [22,23,24,25], silk [26,27], and alginate [28]), their biocompatibility and biodegradability allow tissue regeneration, but they are frequently inadequate with respect to mechanical strength. Conversely, acellular tissue matrices (e.g., small intestinal submucosa (SIS) [10,29,30,31,32,33,34] and decellularized bladder matrix [35,36]), both allogeneic and xenogeneic, can maintain the composition and histological microstructure of the original tissues. The main advantage of these materials is the availability of extracellular matrix (ECM) proteins, such as collagen, elastin, and laminin, and growth factors, which provide a three-dimensional microenvironment optimal for cell growth and organization, enhancing cellular migration and differentiation during regeneration. In fact, the main advantage of these scaffolds consists of the possibility to reconstruct and repair the damaged tissue, signalling host cells to migrate, grow and form new vessels, and to foster differentiation to create specific tissue remodelling. These features are fundamental for a tissue-engineered urinary conduit to enable its considerable larger area to be repopulated with the appropriate cell types, promoting tissue regeneration and proper functionality. For these reasons, it is challenging to recreate the ideal environment for cell migration, growth, and differentiation while preserving tissue properties. An efficient, suitable, non-aggressive decellularization protocol able to preserve ECM components and structure is crucial for the application of decellularized tissues towards regenerative purposes. Another critical key factor is the ability to preserve the tissue mechanical properties while completely removing the remnant DNA fragments from the matrix, which can trigger an inflammatory reaction against the scaffold [37,38].

In the present study, the development of a urinary conduit is proposed, with the aim of assuring a tight urine barrier to protect the stroma and to withstand fluid pressure (physiologic shear stress and radial pressure) [39].

Small intestinal submucosa (SIS) has been found to be a promising biological scaffold due to its biocompatibility, low immunogenicity, high biological activity, and biodegradability; its structure is mainly based on collagen derived from the submucosal layer of porcine small intestine.

Being rich in collagen, proteoglycans, glycoproteins, and growth factors, SIS has been considered an excellent choice for tissue-engineering purposes and clinical applications. Indeed, SIS has the capability to integrate with host tissues and contributes to tissue regeneration, providing an excellent microenvironment for cell adhesion and proliferation, while simultaneously promoting in tissue repair [40].

Therefore, SIS has been extensively used as a scaffold for repairing carotid arteries [41], urinary bladders [29,42], intestinal tracts [43,44], and tendons [45]. The FDA approved the use of SIS in humans for urogenital procedures, such as hernia repair, cystoplasties, ureteral reconstructions, stress incontinence, Peyronie’s disease, penile chordee, and urethral reconstruction for hypospadias and strictures [46,47,48,49,50].

Based on available information and clinical needs, we decided to exploit decellularized SIS to create an impermeable and patent 15-centimeter-long, tissue-engineered urinary conduit with the aim of sparing healthy autologous intestinal segments.

In the case of delicate and thin tissues such as SIS, it is extremely important to establish decellularization and sterilization protocols that must be effective, but non-aggressive for the tissue, in order to preserve the ECM and especially the proteins that enhance tissue regeneration [51].

In this study, we applied a new decellularization protocol by modifying the previously reported TriCol method, which was initially developed for heart valves [52,53]. The decellularized SIS was examined by usual analytical tools (e.g., histology, immunofluorescence, mechanical, and cytocompatibility tests). The protocol was designed for this specific application in order to improve tissue preservation and to replace Triton X-100; its use has been restricted by the European Chemical Agency (ECHA) due to its degradation to a substance with endocrine-disrupting properties [54]. Finally, the decellularization and subsequently sterilization protocols have been optimized by reducing the overall duration and costs [55].

## 2. Results

### 2.1. Decellularization Assessment

**Figure 1:**
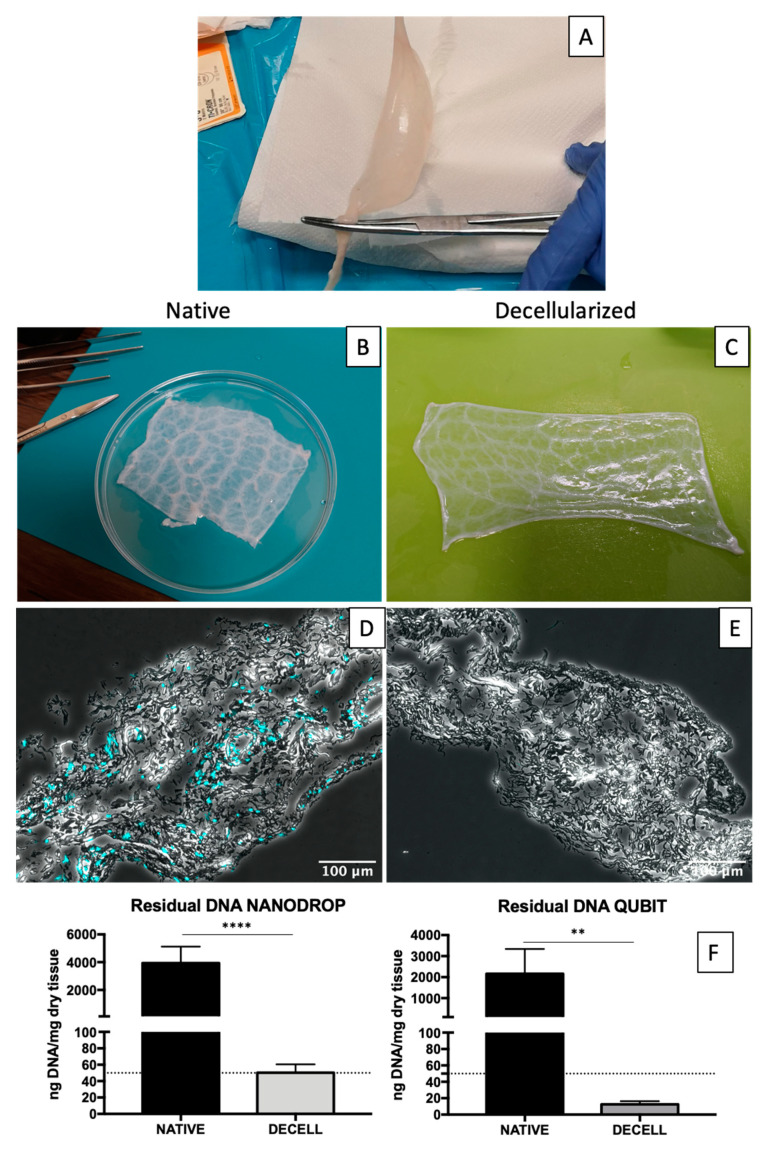
SIS conduit after gentle mechanical removal of the two-external layers of the jejunum (**A**). Native (**B**) and decellularized (**C**) SIS: collagenous fibre bundles are visible through the transparent surface. (**D**) DNA quantification performed in native and decellularized SIS samples: with NanoDrop on the left and with Qubit on the right. Data on graphs show mean ± SD. Data analysed by *t*-test. **** *p* < 0.0001 and ** *p* < 0.01. Merge between phase contrast and DAPI images in case of native (**E**) and decellularized (**F**) shows absence of nuclei in the latter.

Macroscopically, tissue after decellularization process appears well-preserved and similar to native tissue (Figure 1A–C). Microscopically, phase contrast confirmed preservation of fibres after decellularization while nuclei were completely removed (Figure 1D,E).

Two methods have been used for DNA quantification as it is important to accurately evaluate both quality and quantity of nucleic acids in native and decellularized tissue samples: the first measures the UV absorbance at 260 nm (NanoDrop), and the second measures the intensity of a fluorescent dye that specifically binds to double-stranded DNA (Qubit).

DNA quantification revealed a significant reduction in decellularized tissue samples compared to native ones (n = 6) (Figure 1F). With NanoDrop, native SIS presented an average of 3943.60 ± 1175.17 ng DNA/mg dry tissue, whereas for decellularized tissue an amount of 49.94 ± 10.25 ng DNA/mg dry tissue was estimated. With Qubit fluorescence-based quantification, the following values were ascertained: 2158.22 ± 1183.84 ng DNA/mg dry tissue and 12.28 ± 3.94 ng DNA/mg dry tissue for native and decellularized samples, respectively. With both methods, the DNA content in the decellularized SIS was below the threshold of 50 ng dsDNA/mg dry tissue, which is stated as a criterion for successful decellularization [51].

### 2.2. Gross Structure Maintenance

SIS consists of a thin, trilaminate, connective tissue, with a superficial, dense layer of organized collagen (*stratum compactum*) and an underlying, thicker layer of submucosa. The bulk of SIS is composed of loosely and irregularly arranged bundles of collagenous connective tissue with a rich vasculature. The *stratum compactum* appears relatively smooth, while the abluminal surface is more irregular in morphology.

**Figure 2:**
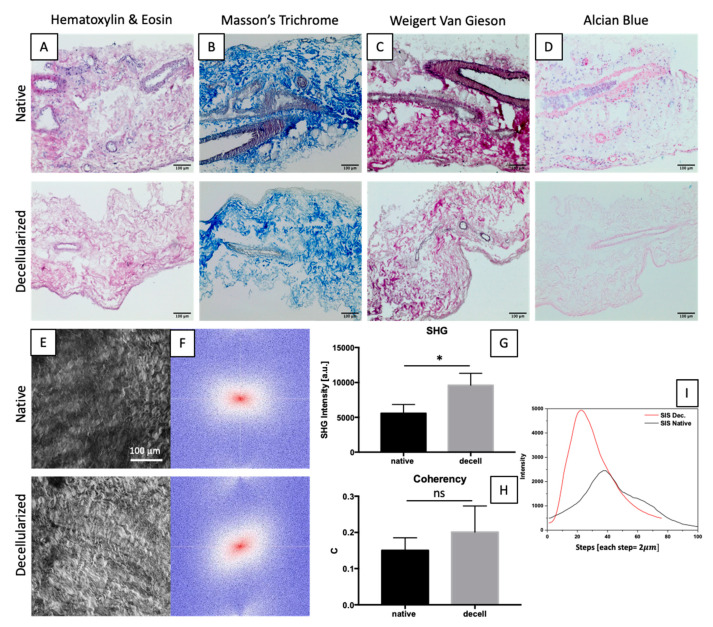
Histological analyses of native and decellularized SIS. H&E shows the effective nuclei removal in decellularized tissue (**A**). Collagen and elastic fibres are revealed by Masson’s Trichrome (**B**) and Weigert–van Gieson (**C**) stainings, respectively. Alcian Blue shows the presence of glycoproteins (**D**). Z-stack of max intensity of phase contrast of native and decellularized SIS (**E**) and representative FFT (**F**). SHG intensity values from z-stack measurements (**G**). Coherency analysis from z-stack (**H**). Data on graphs show mean ± SD. Data analysed by *t*-test. * *p* < 0.05. Representative intensity profile for native and decellularized samples for each step during z-stack acquisition (**I**).

After the decellularization process, the complete removal of nuclei was evident throughout the performed histological analyses. Despite SIS thinness and fragility, the cytoplasmatic component was thoroughly removed, as proved by H&E staining (Figure 2A), while collagen and elastic fibres appeared well-preserved in Masson’s Trichrome and Weigert Van Gieson staining (Figure 2B,C), respectively.

Both native and decellularized SIS were analysed by two-photon microscopy in order to further investigate the preservation of collagen fibres (Figure 2E). The amount of collagen is proportional to SHG intensity (Figure 2G,I), while fibres’ local orientation was evaluated with the coherency parameter (C) (Figure 2H) and FFT (Figure 2F).

We observed a significant increase in SHG intensity following the decellularization process due to a denser distribution of collagen fibres in decellularized tissue. The analysis of the coherency parameter did not highlight any significant change between the two groups, even though the mean coherency value was slightly higher in the decellularized samples. This result was confirmed by the FFT images (Figure 2F).

### 2.3. ECM Maintenance

**Figure 3:**
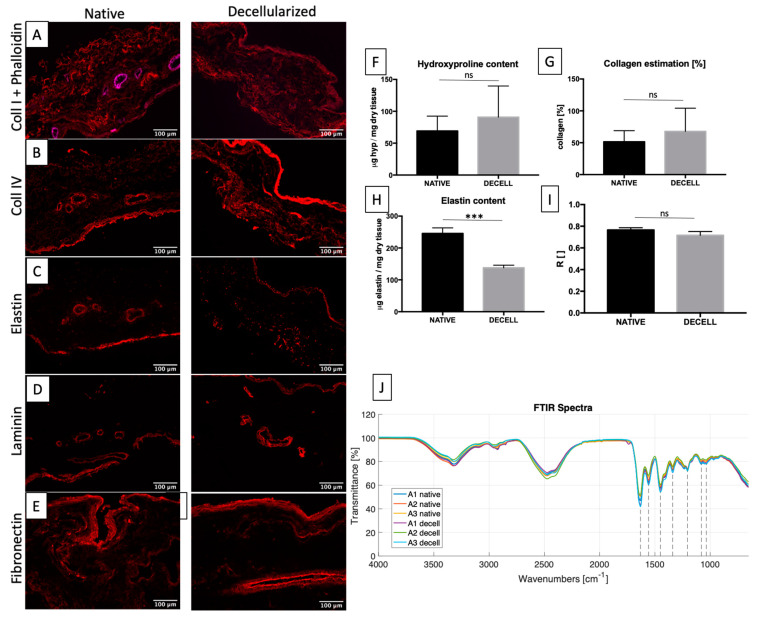
(**A**–**E**): Immunofluorescence of cellular and ECM components: collagen I (red) merged with phalloidin (magenta) (**A**), collagen IV (**B**), elastin (**C**), laminin (**D**) and fibronectin (**E**). Hydroxyproline (**F**) and elastin (**H**) quantifications are reported. No significant difference was found after hydroxyproline quantification, whereas a significant difference was found in the elastin content. Collagen estimation is reported as percentage (**G**), showing no statistical difference by *t*-test. *** *p* < 0.001 and ns *p* ≥ 0.05. The comparison between native and decellularized SIS of the ratio (R) between the peaks related to amide I and II is reported. (No significant difference was found using a *t*-test, *p* ≥ 0.05) (**I**). FTIR spectra are reported, showing the transmittance (%) over the wavenumbers (cm^−1^). Dashed lines highlight peaks at 1631 cm^−1^ (amide I and triple helix), 1558 cm^−1^ (amide II), 1451 cm^−1^ (all types of collagen), 1339 cm^−1^ (collagen I and IV), 1205 cm^−1^ (collagen IV and V), 1080 cm^−1^ (collagen V and VI), and 1035 cm^−1^ (all types of collagen) cm^−1^ (**J**).

Collagen fibres (both type I and IV) were preserved (Figure 3A,B). In comparison to collagen I, collagen IV had a denser distribution close to the surface, corresponding to the *stratum compactum* layer of SIS, and around the vessels. The content of both elastin and laminin, which in native tissue is lower compared to collagen, did not seem to be affected by the decellularization process (Figure 3C,D). While elastin was more abundant in the *stratum compactum* of SIS, laminin was mainly localized around the vasculature.

Hydroxyproline quantification was used to indirectly determine the collagen content in native (n = 6) and decellularized (n = 6) tissue samples: no significant differences were observed (Figure 3F).

Considering that hydroxyproline constitutes 12–14% of collagen and the ratio of hydroxyproline/collagen varies between 7.14–7.69, it is possible to perform an estimation of collagen (coll) in two ways: (i) following the relation given in literature (μg hyp/μm tissue × 7.46 × 100, where 7.46 is the average value on the variation of the ratio hyp/coll) based on the hydroxyproline amount (Figure 3G), and (ii) considering that hydroxyproline constitutes 13.5% of collagen. No significant difference was found between the native and decellularized groups by performing a *t*-test.

Elastin measurement was performed using an elastin assay kit, which is a quantitative dye-binding method for the analysis of mammalian elastin. The elastin amount significantly decreased after decellularization (Figure 3H).

Native and decellularized samples were analysed by FTIR in order to investigate the secondary structure of proteins. Spectra (transmittance vs. wavenumbers) were overlapped in order to show the characteristic peaks and compare the two groups (Figure 3J). Spectra of decellularized tissues were largely overlapped with those of native ones, with the evidence of common peaks at 1631 cm^−1^ (amide I and triple helix), 1558 cm^−1^ (amide II), 1451 cm^−1^ (all types of collagen), 1339 cm^−1^ (specific for collagen I and IV), 1205 cm^−1^ (specific for collagen IV and V), 1080 cm^−1^ (specific for collagen V and VI), and 1035 cm^−1^ (all types of collagen). Characteristic bands include amide I and amide II that arise from the amide bonds between amino acids. The absorption associated with the amide I band (around 1650 cm^−1^) leads to stretching vibrations of the peptide carbonyl group C=O bond of the amide group, while the absorption associated with the amide II (around 1560 cm^−1^) band leads to bending vibrations of the N–H bond. The multiple structures of the collagen (very abundant in SIS) amide I are due to the heterogeneity of its peptide C=O bonds in triple helix, a factor that directly influences the profile of the amide I band infrared spectrum [56,57,58].

In order to avoid artefacts related to the placement of the tissue on the crystal, the ratio (R) between amide I and amide II were calculated (Figure 3I), showing no difference between the native (0.77 ± 0.02) and decellularized (0.72 ± 0.03) groups.

### 2.4. Tensile and Inflation Tests

SIS is a complex bio-composite subjected to multidimensional stresses in vivo [59]; therefore, uniaxial mechanical tests are useful to get a rough estimation of its mechanical behaviour.

**Figure 4:**
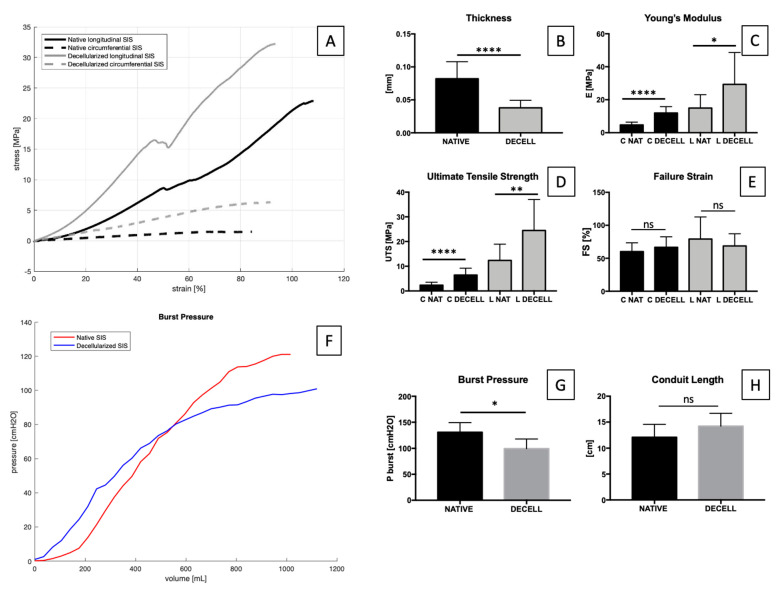
(**A**) Stress/strain curves of native and decellularized SIS along circumferential and longitudinal directions. Thickness, Young’s modulus (**E**), ultimate tensile strength (UTS), and failure strain (FS) are reported correspondingly in (**B**–**E**) comparing native and decellularized tissues. After decellularization, significant differences were found as to thickness, E and UTS, using a *t*-test. No significant (ns) differences were found as to FS values for both directions. Data on graphs show mean ± SD. Data analysed by *t*-test. **** *p* < 0.0001, ** *p* < 0.01 (n = 12) and * *p* < 0.05. (**F**) Burst pressure (cmH_2_O) graph over volume (mL) in native and decellularized SIS. Burst pressure (**G**) and conduit length (**H**) values are reported. Data show mean ± SD (n = 5). Data analysed by *t*-test. *p < 0.05.

Regarding sample thickness, decellularization causes a significant decrease from 0.08 ± 0.03 mm to 0.04 ± 0.01 mm. An increase in stiffness in both circumferential and longitudinal directions was observed after decellularization. Nevertheless, stiffness along the circumferential direction (4.75 ± 1.68 MPa in native vs. 11.97 ± 3.87 MPa in decellularized) was higher than along the longitudinal one (15 ± 8.07 MPa in native vs. 29.33 ± 19.34 MPa in decellularized). Correspondingly, a similar trend was found with regard to UTS values; it can be noticed that the difference between native (12.37 ± 6.60 MPa) and decellularized SIS (24.47 ± 12.56 MPa) along the longitudinal direction is lower than along the circumferential direction (2.34 ± 1.20 MPa in native and 6.45 ± 2.76 MPa in decellularized). This difference was not found with respect to failure strain values, suggesting that the decellularization process could have modified tissue stiffness and elasticity, but not the final elongation before failure (Figure 4A–E).

Due to the configuration of collagen fibres, SIS showed an anisotropic mechanical behaviour both in native and decellularized samples, with higher stresses along the longitudinal direction that represents the preferred fibre orientation. (The total tissue stress is the sum of the single fibres’ stress-strain responses, since the integration of the individual fibres’ mechanical properties and the fibre architecture dictate gross tissue mechanical anisotropy [60]).

For urinary applications, it is also fundamental to assess the tissue’s hydraulic properties since the final tissue-engineered urinary conduit has to assist in preventing urine leakage from the lumen into the surrounding tissues, especially in the case of non-seeded SIS. A relevant property of SIS consists in its sided permeability that is direction-dependent: the mucosal-to-serosal direction is less permeable than is the opposite one.

In order to investigate SIS hydraulic behaviour, a burst pressure test was performed (Figure 4F–H). A significant difference was found between native and decellularized tissue.

### 2.5. Sterility Assessment

The use of normal cell culture medium in direct contact with tissue can be a simple method to detect the presence of contaminants. In the present study, tissue samples were examined in different conditions: native, decellularized, and decellularized plus sterilized with (i) 70% ethanol or (ii) antibiotics/antimycotics and peracetic acid. Following overnight immersion in culture medium, native tissue showed high turbidity with the presence of contaminants, while the other tested groups did not exhibit any change in the pink colour of the media until the end of the observation period (day 5) (Figure S1).

In order to obtain a more complete assessment of sterility, turbidity tests have been performed following an European Pharmacopoeia protocol [61] using thioglycolate and soybean –casein digest media broths. Thioglycollate broth was used to detect the presence of anaerobic bacteria and aerobic bacteria at 35 °C for up to 14 days. Native and decellularized tissues before decontamination showed higher turbidity in all the tubes, while in the case of decellularized plus sterilized with 70% ethanol, only 50% of the tubes were found to be turbid; the remaining tubes were shown to be slightly turbid with a change in broth colour from pink to yellow, opposite of the negative control. In the case of the decellularized plus sterilized (antibiotics/antimycotics and peracetic acid) tissue samples, no change in colour or appearance of turbidity was detected (Figure S1).

In another set of experiments, soybean–casein digest medium broth was used to detect cultures of fungi and aerobic bacteria for up to 14 days at 25 °C. Media from native and decellularized samples exhibited a higher degree of turbidity from day 1 compared to blank, while that from decellularized plus sterilized tissue with 70% ethanol showed some turbidity. In contrast, decellularized tissue sterilized with antibiotics/antimycotics and peracetic acid showed completely clear media upon observation (Figure S1).

Turbidity tests are summarized in Table 1.

### 2.6. In Vitro Cytotoxicity

Decellularized and native tissue samples seeded with human fibroblasts were analysed after 1, 3, and 7 days. Nuclei and cytoskeletons were stained with DAPI and fluorescently labelled phalloidin, respectively. Cells plated on 8-chambered plastic slides were used as a positive control.

**Figure 5:**
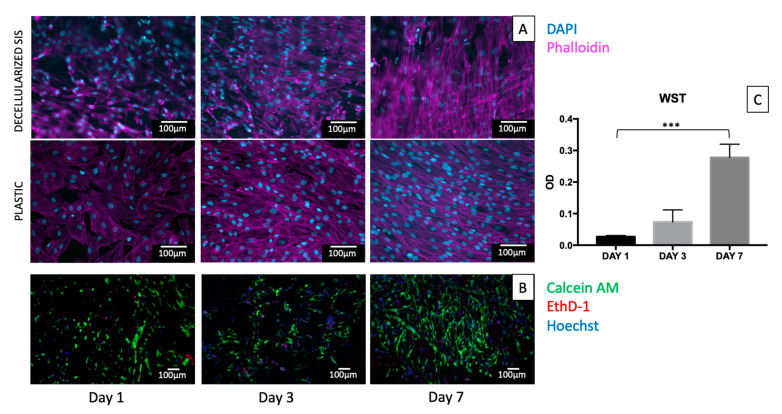
Cell proliferation on decellularized SIS. Patches were seeded with 20,000 cells/cm^2^ and stained with phalloidin (magenta) and DAPI (cyan) (**A**, first row). Control group of cells seeded on plastic is reported in the second row (**A**). Throughout the time course, a progressive increase in cell number was found with a concurrent organization of cells along the principal directions of collagen fibres (here, z-stacks are reported of epifluorescence images). Live/dead staining on seeded SIS at days 1, 3, and 7 (**B**). Nuclei were stained with Hoechst in blue, live cells were stained green with calcein AM. and dead cells were stained in red by ethidium homodimer-1. For all the time points, few dead cells were detected, while an increase in the number of live cells was evident over time, showing also an increase in cell alignment along the SIS fibres. Optical density (OD) on tissue seeded with an initial number of 20,000 cells/cm^2^ is reported (**C**), showing a significant difference between day 1 and day 7. Dunnett’s multiple comparisons test was performed (n = 3), comparing day 3 and day 7 to day 1 (control group). Data show mean± SD, *** *p* < 0.001.

Evident cell proliferation was observed in all tissue samples. In the case of low starting cell densities (10,000 cells/cm^2^), the re-organization of cells on the surface along the collagen fibres of the tissue was observed (Figure S2). With a higher starting cell density (20,000 cells/cm^2^), a strong alignment of actin filaments was visible along the tissue fibres at ±30° with respect to the longitudinal axis of SIS conduit (Figure 5A, first row).

Live/dead staining was performed, and it confirmed the results of fixed cell staining. Calcein AM staining showed a significant increase of the number of viable cells over time from day 1 to day 7. On the other hand, EthD-1 staining for dead cells decreased over this period (Figure 5B).

Cell proliferation was also assessed using the metabolic WST-1 assay at days 1, 3, and 7. The metabolic activity was quantified in decellularized 8 mm diameter patches seeded with human fibroblasts. Significant difference was detected between day 1 and day 7 (Figure 5C).

## 3. Discussion

Radical cystectomy is the gold-standard treatment in the case of urinary bladder cancer; it consists of bladder removal and urinary diversion reconstruction using an autologous intestinal segment. However, the high risk of complications [3,62,63,64] makes it imperative to find an effective alternative to autologous transplantation. Recently, tissue-engineering has been proposed as a helpful solution in the case of tissue and organ substitution [37,38,65]. For a urologic application, the main requirements consist of obtaining a fully impermeable scaffold that must maintain the conduit’s patency, as well as physiological compliance and elasticity. However, the need for a surface impermeable enough for the prevention of urine leakage has to be balanced with features that allow adequate cell migration, adhesion, proliferation, and differentiation [66]. Therefore, the attention was focused on SIS because of its known capability to integrate with host tissues and to promote tissue regeneration, providing the optimal environment for cell adhesion and proliferation [40]. It appears as a thin (~0.1 mm), translucent membrane obtained through the mechanical removal of the *tunica mucosa* from the inner surface and the serosa and *tunica muscularis* from the outer surface [67]. It is a trilaminar portion of the small intestine comprising the *tunica submucosa,* the *tunica muscularis* mucosa and the *stratum compactum* layer of the *tunica mucosa*, which has been reported to be thromboresistant, whereas the opposite surface is thrombogenic [67,68]. Microscopically, the *stratum compactum* layer consists of a denser connective tissue compared to the submucosal layer, but it only comprises 10–15% of the thickness of the tissue [69]. SIS was initially reported as an almost-acellular material, composed of more than 90% of collagen (types I, II, and IV) with 5–10% lipids and a small amount of carbohydrates. It is usually applied as a stacked, multi-layered matrix, and there are many SIS-based commercially available products for use in surgical procedures (e.g., Restore^TM^). However, further studies have shown that this product was not only characterized by highly compacted collagen fibres, but also porcine cells ranging in shape from spindle-shaped (fibroblastic) to round-shaped (possibly mast cells), which was an ultimate cause of inflammatory response after in vivo implantation in mice and rabbits [70]. On the contrary, the results reported in our study assure that decellularized SIS completely lacks native porcine cells, avoiding an eventual adverse response in case of an in vivo implantation.

Previously, several studies tried to assess the usefulness of tubular structures in urinary tract reconstructive urology, and many of them used acellular tissues, and in particular SIS, to create tissue engineered urinary substitutes [66,71]. In the case of urinary diversions, SIS has been used in a rat model, either unseeded or seeded with 3T3 fibroblasts [10]. Porcine SIS was also used by Campodonico et al. [31], who investigated the feasibility of performing UCs, seeding using porcine SIS as a delivery scaffold, in both in vitro and in vivo implantation in a rabbit model. It was found that SIS maintained viability and growth of the surrounding cells in vivo until its degradation.

Moreover, SIS was also applied in urethral reconstruction by Wu et al. [30], who seeded it with UCs and SMCs obtained from differentiated human urine-derived stem cells. Finally, several studies about SIS-based bladder regeneration were carried out in vitro [72], in animal models [29,34,73,74,75,76,77,78,79,80,81,82,83], and in humans [84,85], which included biomechanical evaluations on the regenerated site.

In the present study, a novel decellularization process was applied to porcine SIS with the aim of creating a urinary conduit substitute of 15 cm in length, to be used instead of an autologous intestinal segment after radical cystectomy. To assess the quality of decellularized ECM, four aspects must be assessed: removal of cells, elimination of genetic material, preservation of protein content, and retention of mechanical properties [86].

As widely reported, besides the necessity of preserving ECM, the decellularization process is crucial to prevent an adverse immune response elicited by cell membrane epitopes, residual DNA, and damage-associated molecular pattern molecules (DAMP) [65,87].

The decellularization protocol reported in this study was proven to be effective in the removal of dsDNA: the DNA concentration was found under the threshold of 50 ng/mg dry tissue set by Crapo et al. [51] as necessary to prevent inflammatory reactions caused by animal DNA and the transmission of endogenous retroviruses from animal to patient [88]. In order to increase both specificity and reproducibility of the DNA quantification, it was decided to apply both UV spectroscopy and fluorescence-based methods. (The latter is more sensitive and consistent because it is less influenced by RNA contamination [89,90].) In contrast to commercially available SIS-based scaffolds [70,91] that have been demonstrated as containing measurable amounts of residual DNA before implantation, as in the case of CorMatrix^®^ [92,93], it was possible to achieve an effective removal of DNA residues, reducing the probability of future adverse effects after implantation.

Nevertheless, the relationship between residual DNA amount, fragment length, and immunological reactions following xenotransplantation has yet to be clearly defined, as the biological consequences of residual nuclear material or cytoplasmic debris within the scaffold are still unclear. There are no reports showing a direct cause–effect relation between such cellular remnants and an adverse host response to date [65,88]. Even though the U.S. Federal Drug Administration does not set limits for DNA in biologic scaffolds, more systematic studies are needed in order to determine a more specific threshold in terms of acceptable DNA residues.

Next to effective cellular removal following decellularization, it is just as imperative to preserve ECM, which consists of the secreted products of the resident cells that promote cell attachment, proliferation, and differentiation. However, processes such as decellularization and sterilization may affect the morphology and composition of tissues, including the basement membrane complex [94]. For this reason, it is essential to demonstrate the maintenance of desirable components of the ECM, such as adhesion proteins, including fibronectin, laminin, elastin, and collagens (e.g., collagen I and IV), which would be required for infiltration of the matrix by the cells of choice in vitro or in vivo [65], with the concomitant removal of nuclei.

From a mechanical point of view, together with the muscular layer, SIS plays a major role in providing sufficient structural integrity and strength to the intestine and preventing it from bursting [95], while the other two layers (serosa and mucosa) do not provide a significant contribution. In detail, the porcine jejunum consists of five different layers (from lumen to external side): mucosa, submucosa, circumferential muscle, longitudinal muscle, and serosa. The mucosa layer absorbs nutrients and presents cells that secrete enzymes into the lumen. In the serosa side, the muscle layers are responsible for peristaltic motion, while the serosa membrane surrounds the intestine. The role of SIS is to provide most of the mechanical strength and support vasculature and nerves [59], being very rich in collagen fibres. For this reason, the first response to mechanical stimuli consists in the straightening of the wavy fibres (crimped configuration), while the second response corresponds to the further deformation of the straight fibres. In particular, collagen fibres are distributed biaxially at ±30° with respect to the longitudinal direction with alternating orientation along the thickness. A continuous network of collagen fibres across the intestinal wall gives the intestine structural continuity. The crimped form and the biaxial orientation of collagen act as an elastic sleeve for smooth muscle contractions during the normal peristaltic intestinal activity.

For this reason, it was essential to confirm the preservation of collagen fibres. In this study, two-photon analysis confirmed the preservation of collagen content in porcine SIS after decellularization. The same result has been found indirectly by the FTIR analysis that provides information about the secondary structure of proteins. In our study, the ECM preservation has been demonstrated by overlapping the decellularized and native tissue spectra, with the identification of common peaks related to collagen of type I, IV, V [56], [96,97].

Moreover, hydroxyproline was found to be well preserved in decellularized SIS, and it was used to estimate the collagen content, which was maintained after decellularization. With regard to elastin amount, a decrease was found, causing an increase in tissue stiffness that was also observed in uniaxial tensile tests. (A small increase in stiffness was reported, especially along the circumferential direction, where E and UTS were increased.) The lack of statistical differences in FS demonstrates the maintenance of the final elongation of the tissue. A similar behaviour was seen with hydraulic tests, which were performed to provide insight into the presence and integrity of the structural proteins within the scaffold [65], highlighting slight burst pressure variation between native and decellularized SIS. The results are in accordance with coherency calculation and FFT analysis, where slightly more oriented fibres were found in the case of decellularized tissue.

For the correct interpretation of the obtained data, it is important to remember that connective tissues like SIS are nonhomogeneous, multi-phased materials where each of the components (phases) is organized in a specific structure. The various structures interact with each other during deformation, and the final response of the tissue to the macroscopic deformation is regulated by the interactions between the single components. In detail, the predominant mechanical components are collagen and elastin fibres that are embedded in a matrix (ground surface), which is an amorphous substance, mainly constituted by water and only a low concentration of mucopolysaccharides. From the mechanical point of view, water seems to be the predominant component of the matrix whose viscosity is mainly affected by hyaluronic acid. From the fibres point of view, an important feature is their waviness that is not uniform. For these reasons, the response to mechanical deformations is dual: on the one hand, the fibres rotate and stretch; on the other, the fluid in the matrix is expelled from the fibres under pressure that results from their movement [60].

From a hydraulic point of view, a relevant property of SIS consists in its one-sided permeability that is direction-dependent since the mucosal to serosa side has been previously found to be less permeable than the opposite one. This property has to be taken into consideration in the case of a SIS-based-tissue-engineered urinary conduit: Indeed, it has to be impermeable to prevent urine leakage from the lumen into the surrounding tissues, especially in the case of a non-seeded scaffold. The porosity of SIS is a positive feature that can assist cell-seeding [30], while the impermeability is likely to be further improved once implanted due to subsequent cell ingrowth and new matrix deposition.

Prior to biocompatibility assessment, it was of paramount importance to confirm the efficacy of the sterilization procedure. In fact, cellular scaffolds cannot be directly subjected to sterilization in order to avoid inactivation. Consequently, sterilized acellular scaffolds are then seeded with cells in a clean room environment to ensure sterility in the case of in vivo implant [96].

The ideal scaffold should have the desired properties and should positively interact with cells, including the ability to enhance cell adhesion, growth, migration, and differentiated function [97]. The sterilization procedure consists of the elimination of all forms of living organisms (bacteria, viruses, and yeasts), which can be found in the scaffold, without provoking undesirable changes in the physical and chemical properties of the sterilized material [98,99]. Commonly used sterilization techniques include chemical treatment (ethanol, ethylene oxide), antibiotic treatment, irradiation techniques (ultraviolet irradiation, gamma, and electron beam irradiation), and heat treatment, which have specific advantages and drawbacks.

In the present study, chemical sterilization treatment was chosen in order to guarantee decellularized porcine SIS sterilization. In detail, the use of ethanol was tested due to its low cost and ambient-temperature operating conditions. However, sterility and turbidity tests demonstrated that it is not effective in removing contaminants. The additional concern about denaturation of proteins gave the authors the final reason to avoid the use of ethanol for SIS sterilization. Therefore, both antibiotics/antimycotics and peracetic acid (PAA) based solutions were combined in order to gather their microorganism inactivation ability. While the use of antibiotics is a convenient and simple method of inactivating bacteria by interfering with essential processes, such as DNA replication, cell wall synthesis, and protein synthesis, and being effective against vegetative bacteria and spores, PAA has a relatively high penetration ability that can effectively inactivate a wide variety of microorganisms, including vegetative bacteria, spores, enveloped and naked viruses, and fungi [99]. In this case, sterility tests (culture media did not change in colour and did not become cloudy, and no fungal growth have been found [96]) and turbidity tests (no changes in colour and transparency have been detected [61]) demonstrated the efficacy of this sterilization procedure.

Prior to clinical use, it is important to characterize the potentially harmful effects of the scaffold by cytotoxicity assays. Cytotoxicity means the cascade of molecular events that interfere with macromolecular synthesis, causing distinct cellular, functional, and structural damage. In fact, cells are extremely sensitive to residual chemicals and instantly show signs of toxicity in the presence of potentially harmful substances [100]. In the study, the direct contact assay was chosen in order to study attachment, migration, and distribution of seeded cells within the scaffold. The experiment, repeated with two different starting cells number (10,000 and 20,000 cells/cm^2^), demonstrated SIS cytocompatibility, revealing the enhancement of cell growth during time, with a coarse (10,000 cells/cm^2^) or strong (20,000 cells/cm^2^) live cell organization along SIS fibres, even in depth, respecting the alternate fibres’ orientation along the thickness.

In order to further characterize the cytocompatibility of decellularized SIS, future experiments could evaluate the adhesion and growth of other cell types. Since we were able to demonstrate the maintenance of natural ECM components and cytocompatibility with human fibroblasts (which we suppose would be the main cell type that will repopulate SIS conduit depositing new ECM in case of an in vivo implant), our hypothesis is that our SIS could be biocompatible and bioactive. These are crucial features to guarantee tissue regeneration, promoting cell growth and proliferations.

## 4. Materials and Methods

### 4.1. Porcine Small Intestinal Submucosa (SIS) Procurement

Small intestines were obtained from a local slaughterhouse and treated within 3 hr of the pigs’ sacrifice. The protocols followed by the slaughterhouse were consistent with EC regulations 1099/2009 regarding animal health and protection at the time of slaughter, supervised by the Italian government, and approved by the associated legal authorities of animal welfare (Food and Consumer Product Safety Authority). The preparation of SIS material was done according to the previously described procedure [101,102]. Porcine SIS was obtained through gentle mechanical removal of the two external layers of *tunica muscularis* and *tunica serosa* of the proximal jejunum. The remaining intestine segment (15 cm long) was everted, and the internal *tunica mucosa* was gently abraded with surgical forceps, avoiding perforation through the remaining trilaminate connective tissue layers. The resulting cleaned tissue (100 μm thick) consisted of a translucent tube.

Following the mechanical dissociation, SIS conduits were washed thoroughly with phosphate-buffered saline (PBS) until the solution became transparent.

### 4.2. SIS Decellularization

Porcine SIS was decellularized with an optimized protocol, which included mechanical agitation and the use of protease inhibitors, alternating with hypotonic and hypertonic solutions. We incubated tissue with a non-ionic detergent (Tergitol, 15S9, Sigma-Aldrich, Saint Louis, MO, USA) and an ionic one (Sodium Cholate, C1254, Sigma-Aldrich, Saint Louis, MO, USA) with intermediate washing cycles in PBS or saline solution, with a subsequential alcohol-based solution for tissue decontamination and delipidization, and endonuclease (Benzonase^®^, E1014, Sigma-Aldrich, Saint Louis, MO, USA) treatment to remove the residual DNA fragments.

The resulting acellular tissue was treated with two different protocols (the first based on 70% ethanol/water and the second based on an antibiotic/antimycotic plus peracetic acid solution) to sterilize it in preparation for cytocompatibility tests.

### 4.3. DNA Quantification

The total DNA content was extracted from samples of lyophilized native (5–10 mg) and decellularized SIS (7–15 mg) using a DNeasy Blood & Tissue Kit (69506, Qiagen, Valencia, CA, USA). Concentration was measured at 260 nm with a NanoDrop One Spectrophotometer (Thermo Scientific, Waltham, MA, USA) and with a Qubit 2.0 (Thermo Fisher Scientific, Waltham, MA, USA), using a Qubit^TM^ 1X dsDNA HS Assay Kit (Q33231, Thermo Fisher Scientific, Waltham, MA, USA). Total DNA values were normalized to the dry weight.

### 4.4. Histological Stainings

Fixed native and decellularized tissue samples were frozen in liquid nitrogen with optimum cutting temperature (OCT) compound. Cryosectioned tissue slices (6 μm thick) were used for the histological analyses using a Hematoxylin and Eosin kit (04-061010, BioOptica, Milan, Italy) and Masson’s Trichrome (04-010802, BioOptica, Milan, Italy) to assess the presence of nuclei (black), collagen (blue), cytoplasm and muscle fibres (red); Weigert–van Gieson (long method, 04-051802, BioOptica, Milan, Italy) to display elastic fibres, connective tissue, collagen, and nuclei (black); an Alcian Blue Stain Kit (pH 2.5, Mucin Stain, ab150662, Abcam, Cambridge, UK) for the visualization of sulphated and carboxylated acid mucopolysaccharides and sulphated and carboxylated sialomucins (glycoproteins). Each procedure was performed according to the supplier’s instructions.

Images were acquired with an EVOS XL Core Cell Imaging System (Thermo Fisher Scientific, Waltham, MA, USA).

### 4.5. Immunofluorescence

In order to evaluate the presence/absence of nuclei and to analyse ECM composition and structural integrity, immunofluorescence was performed on cryosectioned samples. SIS sections (6 μm thick) were fixed in 4% *v*/*v* paraformaldehyde (PFA) for 5 min and then washed thrice with PBS. Slices were then blocked with 1% *w*/*v* bovine serum albumin (BSA) for 30 min at RT. Afterwards, slides were incubated with antibodies raised against collagen I (1:100, C2456, Sigma-Aldrich, Saint Louis, MO, USA), collagen IV (1:200, ab6586, Abcam), elastin (1:50, ab21610, Abcam), and laminin (1:200, L9393, Sigma-Aldrich, Saint Louis, MO, USA) overnight at 4 °C. After washing with PBS, slides were incubated with Alexa Fluor 555 goat anti-mouse IgG (1:300, A21422, Thermo Fisher Scientific, Waltham, MA, USA) and goat anti-rabbit IgG (1:300, A27039, Thermo Fisher Scientific, Waltham, MA, USA) secondary antibodies as well as with phalloidin—Atto 647N (1:200, 65906 Sigma-Aldrich, Saint Louis, MO, USA) for 1.5 h at RT. Lastly, DAPI (NucBlue Fixed Cell Stain ReadyProbes reagent, R37606, Thermo Fisher Scientific, Waltham, MA, USA) was used to stain nuclei.

For whole-mount immunofluorescence, patches were fixed in 4% *v*/*v* PFA for 5 min at RT, permeabilized with 0.1% *v*/*v* Triton X-100 for 15 min, followed by blocking in 1% *w*/*v* BSA solution for 30 min. Samples were then incubated with phalloidin—Atto 647N (1:200) and DAPI to stain F-actin filaments and nuclei, respectively.

Images were acquired with an epifluorescence microscope Leica AF6000 connected to a Leica DC300 digital camera and equipped with LAS AF Software (Leica Micro-Systems, Wetzlar, Germany). Post-imaging analysis was performed using ImageJ software.

### 4.6. Two-Photon Microscopy

Native and decellularized SIS samples (tissue patches) were fixed in 4% PFA in PBS (Bioptica, Milan, Italy) for 20 min at RT, and then stored in PBS at 4 °C until analysis. SIS native and decellularized patches were analysed with two-photon microscopy to assess the impact of the decellularization process on the collagen structure by measuring second harmonic generation (SHG) signal [103].

Two-photon analysis was performed using a custom-made multimodal microscope [104]. Briefly, an incident wavelength of 800 nm was adopted to detect the collagen’s SHG signal at 400 nm. Images were acquired at fixed magnification through the Olympus 25X water immersion objective with 1.05 numerical aperture (1024 × 1024 pixels), averaged over 70 consecutive frames, with a pixel size of 0.43 μm. Multiple z-stacks from native and decellularized SIS were recorded with the same acquisition settings, therefore being directly comparable with each other.

For quantitative measurements, the RAW uncompressed images were analysed by using ImageJ software [105]. We considered SHG intensity as an indicator of total collagen content. Then, we evaluated collagen fibre distribution considering the fast Fourier transformation (FFT) that converts the image into a new form using the spatial frequency properties of the pixel intensity variations, and the coherency (C) parameter, to estimate the local orientation of the fibres (values around 0 indicate isotropic areas, whereas values around 1 indicate highly oriented structure), using the plug-in OrientationJ [106,107].

### 4.7. Mechanical Tests

Sample thickness was measured using a Mitutoyo digital calliper (model ID-C112XB, Aurora, Illinois, USA) and mean and standard deviation values were calculated. Uniaxial tensile loading tests were performed by means of a custom-made apparatus (IRS, Padova, Italy); it consists of four linear actuators and loading cells (50 N) and the software interface was implemented in LabVIEW. Samples were cut with a custom-made cutter into dog-bone shaped specimens, according to the ASTM D1708-13 standard concerning small-size tissues, with a gauge length of 5 mm and width of 2 mm. Tests were performed at room temperature, and samples were kept hydrated with 0.9% NaCl solution. Samples were pre-loaded up to 0.1 N, then elongated (elongation rate 0.2 mm/s) to rupture to measure ultimate tensile strength (UTS) and failure strain (FS) and to calculate Young’s modulus (E) as the slope of the stress-strain curves in the linear region. Engineering stress (MPa) was calculated as the tensile force measured by the loading cells (N) divided by the original cross-sectional area of the sample. The strain (%) was defined as the ratio between the grip displacement and the gauge length. SIS samples were tested along the circumferential and longitudinal directions.

Biomechanical parameters were calculated from the stress-strain curves with an in-house-developed Matlab^®^ script (Mathworks, Natick, MA, USA).

### 4.8. Inflation Tests

Two rubber tubes (external diameter of 8.4 mm) were put through the tubular sample and fixed by rubber bands and electrical cable ties. The length of the sample was collected starting from the end of the tubes in order not to consider the part that wraps the tubes. A peristaltic pump (VerderFlex Vantage 5000, Verder Ltd., Castelford, UK) and a pressure transducer (142 pc 01d, Honeywell, Charlotte, NC, USA) were connected to the sample by rubber tubes. The transducer was interfaced to a laptop PC by a microcontroller (Arduino MEGA 2560, Arduino LLC, USA). Data storage was set at 5 Hz sampling rate. The sample was then completely immersed in saline solution to prevent gravity effects. The inflation tests were performed by imposing a continuous flow (velocity of 25 mL/s) up to the rupture of the structure, visible by means of real-time pressure recording on a PC screen. The rupture is detected by a pressure peak followed by a dramatic decrease until the recorded pressure is equal to zero (environmental pressure).

### 4.9. FTIR Analysis

Native (n = 3) and decellularized (n = 3) porcine SIS tissues were processed for Fourier transform infrared spectroscopy (FTIR) using a Nicolet iS-50 spectrometer (Thermo Fisher Scientific, Waltham, MA, USA) with an Attenuated Total Reflectance (ATR) accessory. The instrument was equipped with diamond/ZnSe crystal and pressure arm. Infrared spectra of the samples and background were collected using 64 scans in the range of 4000-500 cm^−1^ to characterize the composition of each group of samples.

Data were analysed using a Matlab^®^ script (Mathworks, Natick, MA, USA) [108].

### 4.10. Protein Analysis

#### 4.10.1. Elastin Quantification

Elastin quantification was performed with a Fastin Elastin Assay kit (F2000, Biocolor, Carrickfergus, UK). Briefly, lyophilized native and decellularized tissues were treated with 0.25 M oxalic acid at 100 °C for 3 h (3 extractions of 1 h each) to extract α-elastin. The three consecutive extractions were pooled, and elastin was precipitated by incubating with elastin precipitating reagent for 15 min at RT. To enhance precipitation of acid soluble α-elastin, dye reagent was added to all samples, followed by centrifugation; dye reagent was mixed by vortex and placed for 90 min on a mechanical shaker to allow the reaction between the α-elastin and the dye. After centrifugation at >10,000× *g*, supernatant was drained, and dye dissociation reagent was added and thoroughly mixed to ensure the binding of dye with all available elastin. The absorbance was measured at 513 nm in a microplate reader (Spark 10M, Tecan, Mannedorf, Switzerland). Standard curves were plotted using α-elastin standards. Elastin content was expressed as μg elastin/mg dry tissue.

#### 4.10.2. Hydroxyproline Quantification

Hydroxyproline quantification was performed with a Hydroxyproline Assay Kit (MAK008, Sigma-Aldrich, Saint Louis, MO, USA), following the manufacturer’s instructions. Briefly, lyophilized native and decellularized SIS samples (≈5 mg lyophilized dry tissue) were homogenized in equal volumes of water and hydrochloric acid (12 M) and hydrolysed at 120 °C for 3 h. The supernatant of each sample was transferred to a 96-well plate and evaporated in an oven at 60 °C. Chloramine T/oxidation buffer mixture was added to each sample and incubated for 5 min at RT. Then, diluted DMAB reagent was added and incubated at 60 °C for 90 min. Absorbance was measured at 560 nm with a Spark 10M microplate reader (Tecan, Mannedorf, Switzerland). Standard curves were generated using a hydroxyproline standard solution provided by the manufacturer. Hydroxyproline content was calculated as μg hydroxyproline/mg dry tissue.

Based on the hydroxyproline quantity, the collagen content was estimated according to two methods: (i) $collagen\;\left[\%\right]=\frac{g\;\mathrm{hydroxyproline}\;}{g\;\mathrm{dry}\;\mathrm{tissue}\;}*7.46*100$, where the value 7.46 is the average value of the range 7.14 – 7.69 in which the hydroxyproline/collagen ratio varies; and (ii) $hydroxyproline\;\left[\frac{\mu g}{mg}\right]=13,5\%\;collagen\;\left[\frac{\mu g}{mg}\right]$ [109,110].

### 4.11. SIS Sterilization

Samples of decellularized tissue were subjected to sterilization procedures comparing the efficacy of two different protocols. In the first protocol, tissue samples were treated with 70% ethanol for 30 min at room temperature, while in the second, a cocktail of antibiotics and antimycotics (50 mg/L vancomycin hydrochloride, 8 mg/L gentamicin, 240 mg/L cefoxitin, and 25 mg/L amphotericin B) was applied at 37 °C for 24 h followed by 3 hr treatment with 0.1% of peracetic acid (PAA) [111].

### 4.12. Sterility Assessment

The level of sterility was investigated by two qualitative procedures to assess the absence of bacterial and fungal contamination. The 8 mm diameter punches of test samples (each in duplicate) of native, decellularized, and eventually sterilized tissues were immersed in two different sets of culture medium.

Briefly, in the first qualitative method, two punches from each tissue were immersed in T25 tissue culture flasks containing RPMI media with 10% *v*/*v* FBS (20 mL). The flasks were kept in an incubator at 37 °C for up to 5 days. The contamination was observed by the appearance of turbidity after 24–96 h. Images were recorded periodically.

In the second protocol, the patches were treated according to the standardized guidelines of the European Pharmacopoeia [61]. Two different broths, thioglycollate medium (cat no. T9032, Sigma-Aldrich, Saint Louis, MO, USA) and soybean–casein digest medium (cat no. 22092, Sigma-Aldrich, Saint Louis, MO, USA), were used to detect aerobic/anaerobic bacteria and fungi. The broths were incubated at 35 °C and RT for 14 days, respectively. In all the four groups (native, only decellularized, decellularized, and sterilized with ethanol or with AA/PAA) turbidity was evaluated through visual inspection and imaging was performed periodically.

### 4.13. In Vitro Cytotoxicity Assays

In vitro cytotoxicity of decellularized tissue samples was evaluated qualitatively and quantitatively by a contact assay, in accordance to part 5 of ISO 10993 on the biological evaluation of medical devices [112]. Decellularized and sterilized SIS samples were cut in 2 × 2 cm^2^ squared specimens and fit into custom-made inserts (I.D. 8 mm) in aseptic conditions. Inserts were placed in 24-well plates to seed cells on the exposed side of tissue.

Prior to cell seeding, samples were incubated with DMEM- (high glucose) containing 20% FBS and 1% Penicillin–Streptomycin at 37 °C. Human fibroblasts (BJ cell line) were seeded on the samples at two different densities (10,000 cells/cm^2^ and 20,000 cells/cm^2^) and cultured for 1 day, 3 days, and 7 days using the aforementioned media. Cell proliferation and viability was assessed by immunofluorescence, live/dead staining, metabolic proliferation assay, and dsDNA quantification.

### 4.14. Live/Dead Assay

Cell viability was evaluated using the Live/Dead viability/cytotoxicity kit (MP 03224, Thermo Fisher Scientific, Waltham, MA, USA). Live cells were stained in green by calcein AM (2 µM), while dead cells in red by Ethidium homodimer-1 (4 µM). Additionally, nuclei were stained in blue with Hoechst 33258 (Sigma-Aldrich, Saint Louis, MO, USA). Seeded samples were incubated at 37 °C for 45 min. At the end of the incubation period, the staining solution was removed, and fresh medium was added to take epifluorescence images by means of an Olympus IX71 microscope.

### 4.15. WST Assay

A WST-1 Cell Proliferation and Cytotoxicity Assay kit (AR1159, Boster, Pleasanton, CA, USA) was used to assess viability and proliferation of cells cultured on decellularized patches at the end of each time point. This assay indirectly measures the activity of the enzyme NADPH oxidase through the reduction of WST-1 reagent into the orange/yellow, water-soluble formazan molecule. The concentration of this product is directly proportional to the metabolic activity of cells. Absorbance was read at 450 nm with a microplate reader (Spark 10M Tecan, Tecan, Mannedorf, Switzerland). The fluorescence values of the no-cell control wells were averaged and subtracted from the fluorescence value of each experimental well.

## 5. Conclusions

SIS has shown to be a promising scaffold for tissue-engineering applications. The new optimized decellularization protocol allowed the improved preservation of the tissue with an optimal removal of cells and DNA remnants. Moreover, the used sterilization protocol was proven effective and safe for subsequent cell seeding. Cytocompatibility tests using fibroblasts showed promising results as cells were able to attach and proliferate over time on decellularized SIS, infiltrating into it and aligning with its collagen fibres.

Further analyses should be performed with other cell types (e.g., endothelial and smooth muscle cells differentiated from MSCs), considering the specific clinical need to obtain an impermeable cellular layer, without using autogenous UCs due to their malignant potential in urinary bladder cancer applications. Moreover, a possible tissue reinforcement could be necessary in order to increase SIS-conduit patency and impermeability, e.g., coupling the already-tested decellularized SIS with a polymer, creating a hybrid membrane that could improve the mechanical properties of the urinary conduit while exploiting SIS cytocompatibility.

## Figures and Tables

**Table 1:** Summary of turbidity tests performed in native, only decellularized, and decellularized treated with 70% ethanol or antibiotics/antimycotics/PAA, compared with control group (only media).

Sample Name	Turbidity within 14 Days (Yes/NO)
Native	YES
Only decellularized	YES
Decellularized + 70% Ethanol	YES
Decellularized + antibiotic/antimycotic/PAA	NO
Control (only media)	NO

## Data Availability

Not applicable.

## Supplementary Materials

The following supporting information can be downloaded at: https://www.mdpi.com/article/10.3390/ijms23052826/s1.
