# Supplementary material for: Porcine Small Intestinal Submucosa (SIS) as a Suitable Scaffold for the Creation of a Tissue-Engineered Urinary Conduit: Decellularization, Biomechanical and Biocompatibility Characterization Using New Approaches
Source: Int J Mol Sci. 2022 Mar 4;23(5):2826. doi: 10.3390/ijms23052826 (PMC8910833; doi:10.3390/ijms23052826)
Supplement: Supplementary file 1 [file ijms-23-02826-s001.zip › ijms-1616817-supplementary.pdf]

Supplementary

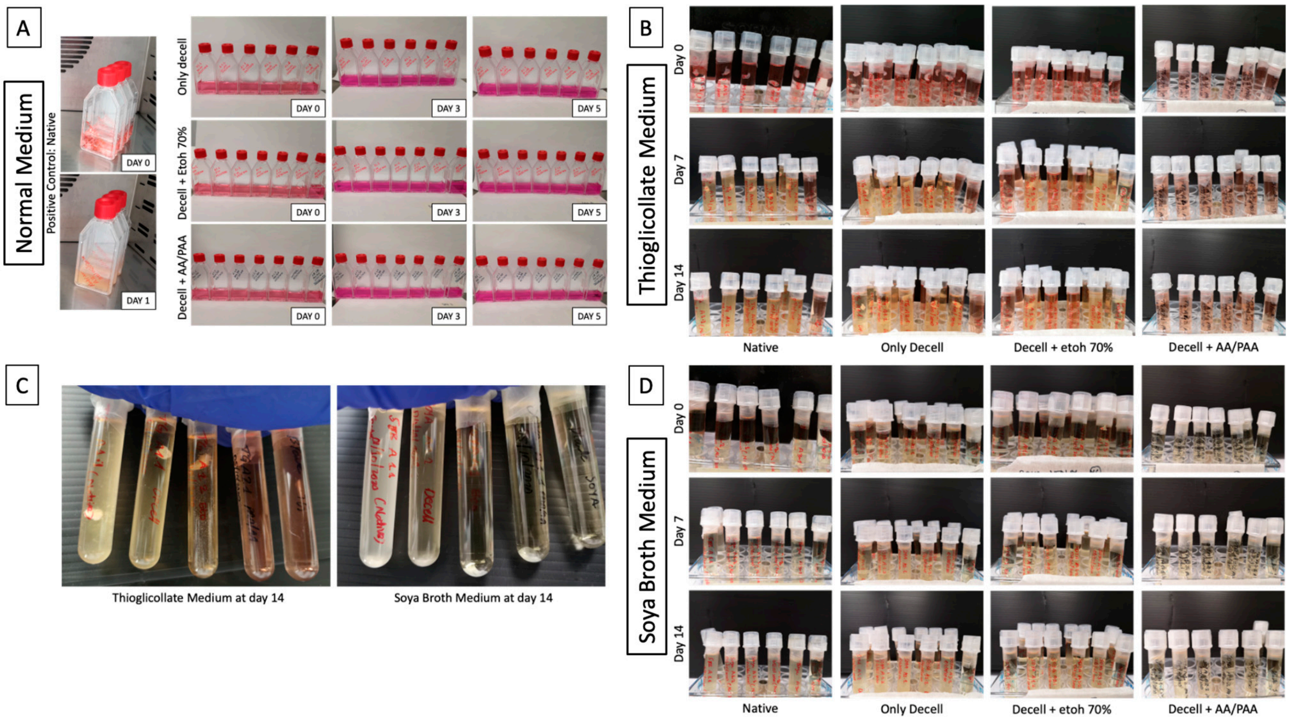

Figure S1: Sterility test with normal medium (A) and turbidity tests with Thioglycolate medium (B) and Soya Broth medium (D). Zoom of day 14 of turbidity tests is reported (C).

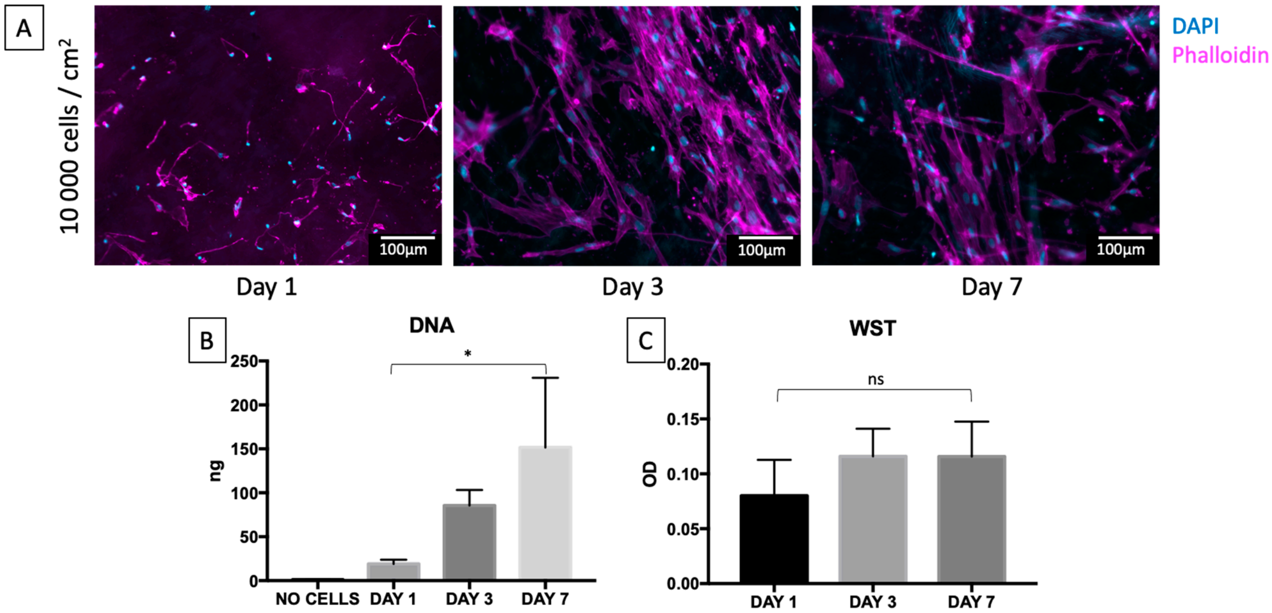

*Figure S2: Cell proliferation on decellularized SIS. Patches were seeded with 10 000 cells/cm² and stained with phalloidin (magenta) and DAPI (cyan) (A). DNA (B) and WST (C) graphs are reported below. Significant difference was found between day 1 and day 7 in DNA where Tukey 's multiple comparisons test was performed, *p<0,05 (n=3). No differences were found in WST graph performing Dunnett's multiple comparisons test (n=3). Data show mean± SD.*
